# Comparative Analysis of Fluorescence In Situ Hybridization and Next-Generation Sequencing in Sperm Evaluation: Implications for Preimplantation Genetic Testing and Male Infertility

**DOI:** 10.3390/ijms252011296

**Published:** 2024-10-21

**Authors:** Efthalia Moustakli, Antonios Gkountis, Stefanos Dafopoulos, Athanasios Zikopoulos, Sotirios Sotiriou, Athanasios Zachariou, Konstantinos Dafopoulos

**Affiliations:** 1 Laboratory of Medical Genetics, Faculty of Medicine, School of Health Sciences, University of Ioannina, 45110 Ioannina, Greece; thaleia.moustakli@gmail.com; 2 Genesis Athens Thessaly, Centre for Human Reproduction, 41335 Larissa, Greece; antmagkountis@gmail.com; 3 Department of Health Sciences, European University Cyprus, 2404 Nicosia, Cyprus; stefanosntf2001@gmail.com; 4 Royal Devon and Exeter Hospital, Barrack Rd., Exeter EX 25 DW, UK; thanzik92@gmail.com; 5 Department of Embryology, Faculty of Medicine, School of Health Sciences, University of Thessaly, 41110 Larissa, Greece; sotiriousot@yahoo.gr; 6 Department of Urology, School of Medicine, Ioannina University, 45110 Ioannina, Greece; zahariou@otenet.gr; 7 Department of Obstetrics and Gynecology, Faculty of Medicine, School of Health Sciences, University of Thessaly, 41110 Larissa, Greece

**Keywords:** fluorescent in situ hybridization, PGT-A, chromosomal abnormalities, sperm evaluation, embryo selection, genetic screening, reproductive outcomes, male infertility

## Abstract

Pre-implantation genetic testing (PGT) is a crucial process for selecting embryos created through assisted reproductive technology (ART). Couples with chromosomal rearrangements, infertility, recurrent miscarriages, advanced maternal age, known single-gene disorders, a family history of genetic conditions, previously affected pregnancies, poor embryo quality, or congenital anomalies may be candidates for PGT. Preimplantation genetic testing for aneuploidies (PGT-A) enables the selection and transfer of euploid embryos, significantly enhancing implantation rates in assisted reproduction. Fluorescence in situ hybridization (FISH) is the preferred method for analyzing biopsied cells to identify these abnormalities. While FISH is a well-established method for identifying sperm aneuploidy, NGS offers a more comprehensive assessment of genetic material, potentially enhancing our understanding of male infertility. Chromosomal abnormalities, arising during meiosis, can lead to aneuploid sperm, which may hinder embryo implantation and increase miscarriage rates. This review provides a comparative analysis of fluorescence in situ hybridization (FISH) and next-generation sequencing (NGS) in sperm evaluations, focusing on their implications for preimplantation genetic testing. This analysis explores the strengths and limitations of FISH and NGS, aiming to elucidate their roles in improving ART outcomes and reducing the risk of genetic disorders in offspring. Ultimately, the findings will inform best practices in sperm evaluations and preimplantation genetic testing strategies.

## 1. Introduction

Early human embryos frequently exhibit chromosomal aneuploidy, which can negatively impact the effectiveness of assisted reproductive technology (ART). Aneuploidy affects up to 60% of early pregnancy losses, 4% of stillbirths, and 0.3% of live births [1]. The presence of extra chromosomes in an embryo can hinder its development and lead to complications, including the possibility of miscarriage. Quantifying the percentage of sperm or oocytes exhibiting chromosomal abnormalities may provide valuable insights into reproductive potential and genetic risks [2]. Next-generation sequencing (NGS) is the most widely used method for comprehensive prenatal genetic testing (PGT). NGS is useful for selecting embryos with euploid chromosomes and no harmful mutations by analyzing embryos generated through various methods, including but not limited to intracytoplasmic sperm injection (ICSI) treatment [3].

Accurate gene transmission relies on the integrity of sperm DNA. Recent data show a significant correlation between sperm DNA damage and an increased risk of miscarriage following ICSI and in vitro fertilization (IVF). When reproductive prospects are unfavorable, examining sperm chromosomes can aid in diagnosis and treatment planning [4,5]. Preimplantation genetic testing for aneuploidies (PGT-A) is particularly relevant for certain patient groups, as chromosomal defects in gametes can be passed on to embryos [3]. Even in cases where the female partner is under 36 years of age, embryos from asthenozoospermic patients may exhibit a significant frequency of chromosomal abnormalities [6]. Severe male factor infertility may counteract the maternal influence that generally leads to the development of euploid embryos in natural conceptions. Cytogenetic sperm analysis is used to study the paternal contribution to implantation failure and recurrent miscarriage. While the majority of chromosomal anomalies are attributed to maternal factors, 8–12% of abortions involving trisomy of chromosomes 12, 18, and 21 are linked to paternal contributions [7,8]. Despite the available data, there is ongoing debate regarding the clinical use and reproductive benefits of fluorescence in situ hybridization (FISH). This review aims to compare and evaluate the FISH method for sperm evaluation against the NGS approach, highlighting both its benefits and limitations [9].

## 2. Fluorescent in Situ Hybridization (FISH)

Aneuploidy, defined as deviations from the standard haploid or diploid chromosomal complement, is assessed using the cytogenetic technique of fluorescence in situ hybridization (FISH). While FISH primarily identifies numerical abnormalities, the chromosomal integrity of sperm also involves the assessment of possible structural abnormalities, which may require additional specific tests [10]. Since infertile gametes have a higher incidence of chromosomal abnormalities compared to those from fertile men, a comprehensive clinical diagnosis requires an assessment of all human autosomes and sex chromosomes. Advanced and costly laboratory assays, such as amniocentesis, chorionic villus sampling (CVS), karyotyping, fluorescence in situ hybridization (FISH), non-invasive prenatal testing (NIPT), quantitative PCR (qPCR), and microarray analysis, are frequently employed to assess specific chromosomal abnormalities, particularly viable trisomies of chromosomes 13, 18, and 21. These conditions can lead to live births but often come with serious health implications. Additionally, these assays can also evaluate non-lethal chromosomal conditions such as X monosomy and Klinefelter syndrome. The advanced technologies employed, the medical procedures necessary for sample collection, and the skill required for result analysis and interpretation are all factors in the high cost of these assays [6,11].

Aneuploidy can result from nondisjunction, anaphase lag, or inefficient checkpoint management, with the most critical abnormalities involving whole chromosome losses or gains [12]. Structural chromosomal changes become more frequent with advancing paternal age. Data from studies indicate that older mice have increased gonosomal disomy (X-X-8), suggesting a possible link between meiotic abnormalities and advanced paternal age. Since aneuploidy cannot be identified through simple microscopic observation, andrology labs typically employ advanced techniques like karyotyping and fluorescence in situ hybridization (FISH) to accurately detect conditions such as trisomy [13]. In prenatal testing, more invasive procedures like amniocentesis and chorionic villus sampling (CVS) are used.

Sperm FISH analysis provides a simpler yet effective method for assessing male gametes. Prior to analysis, the compacted chromatin in the sperm head must be addressed to allow decondensation. Multicolor FISH is utilized to determine the frequency of aberrant sperm [9,10].

Advanced image analysis software and a large sample size are necessary for accurate results. However, FISH has limitations, such as its inability to detect entire chromosomes and survey structural chromosomal aberrations [14]. Despite these drawbacks, FISH remains the most practical and efficient method for evaluating sperm aneuploidy in andrology labs. The procedure includes material processing, decondensation, hybridization, post-hybridization washes, and visualization [10,15]. Strict criteria are used in chromosomal aneuploidy assessment to minimize subjectivity. Sperm cells with unclear borders or overlapping with other cells are excluded from the count. Additionally, cells with missing chromosomes (nullisomies) and those with extra chromosomes (disomies) are both considered forms of aneuploidy, but they are analyzed separately due to their distinct impacts on fertility outcomes [12].

## 3. FISH Analysis Indications

The primary goal of fluorescence in situ hybridization (FISH) is to evaluate the chromosomal integrity of sperm cells, which can provide insights into male fertility and potential genetic risks in offspring. FISH uses fluorescently labeled DNA probes that bind to specific chromosomes or chromosomal regions, allowing for the identification of abnormalities such as trisomy (extra chromosome) or monosomy (missing chromosome) [16]. Sperm aneuploidy can contribute to infertility, and its associated risk factors include birth defects and recurrent implantation failure (RIF). By screening for genetic abnormalities, FISH can provide insights into reproductive outcomes [10].

Cytogenetic sperm analysis is valuable for identifying candidates for assisted reproductive techniques. Research indicates that sperm aneuploidy is linked to decreased sperm motility, making FISH a useful tool for semen evaluation [12,17]. Men with normal semen and women experiencing repeated miscarriages can benefit from FISH analysis, which may also help assess the likelihood of having genetically defective offspring [10].

Chromosome abnormalities are more common in infertile individuals, with chromosomal aberrations occurring regardless of sperm count. Testicular sperm may be less suitable for intra-cytoplasmic sperm injection (ICSI) due to higher aneuploidy rates in chromosomes 13, 18, 21, X, and Y [6,18]. Men with oligoasthenoteratozoospermia (OAT) or XXY/XY mosaics face an increased risk of miscarriage due to the production of 24,XY spermatozoa, which can lead to the conception of 47,XXY embryos that have a low probability of survival [19]. Studies have shown a significant increase in sex chromosome and autosomal disomy in patients with OAT. There may be a threshold effect where a higher frequency of sperm aneuploidy increases the likelihood of chromosomal abnormalities in embryos. XXY and XYY karyotypes are among the most common chromosomal abnormalities observed in infertile men. For cases where no spermatozoa are present in the ejaculate, operative sperm extraction is an alternative approach [11,20].

Reduced sperm production due to chromosomal abnormalities can lead to decreased sperm count. Men with nonobstructive azoospermia are more likely to have sperm cells with an abnormal chromosomal number (aneuploidy), and in some cases, sperm cells may have diploid nuclei. Changes in genetic regulation during mitotic cell division and proliferation [21] and underlying regulatory mechanism abnormalities may contribute to a higher prevalence of malformations. Couples with repeated miscarriages often have sperm parameters within normal ranges, complicating the grouping of patients for FISH investigations due to the diverse clinical presentations associated with these defects [22].

## 4. Recurrent Abortion and Implantation Failure Are Associated with Aneuploidy in Spermatozoa

**Figure 1:**
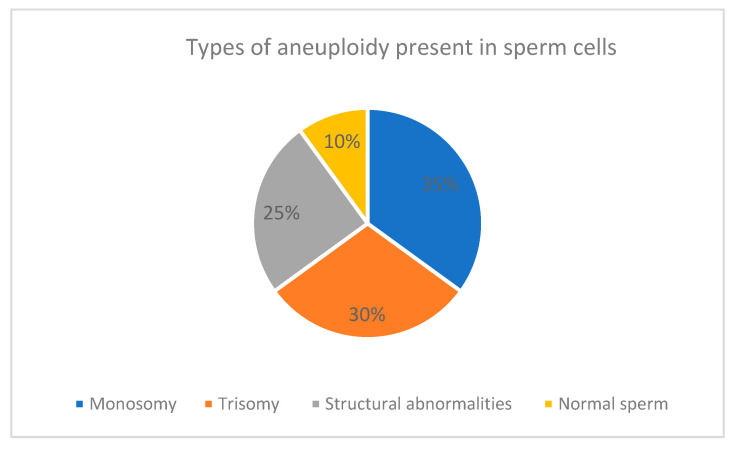
This pie chart illustrates the distribution of aneuploidy types in spermatozoa, highlighting the proportions of normal spermatozoa, structural chromosomal abnormalities, monosomy, and trisomy.

Spermatozoa undergo a complex developmental process called spermatogenesis. During this process, errors can lead to chromosomal abnormalities (Figure 1). Aneuploidy refers to an abnormal number of chromosomes within a cell, resulting from improper chromosome distribution during cell division or issues in meiosis, where homologous chromosomes or sister chromatids fail to segregate correctly [6,23]. This results in cells with extra or missing chromosomes [24]. When such aneuploid spermatozoa fertilize an oocyte, it can lead to various reproductive complications, including spontaneous abortion (miscarriage) and recurrent implantation failure (RIF) [25]. Recurrent abortion is defined as two or more consecutive pregnancy losses before 20 weeks of gestation. Aneuploid spermatozoa are a significant risk factor for this condition because chromosomal abnormalities often result in embryonic or fetal malformations that are incompatible with life [26,27].

Sperm aneuploidy is more common in males with a history of adverse reproductive outcomes, such as recurrent abortions or failures after intra-cytoplasmic sperm injection (ICSI). Despite having a normal 46,XY karyotype, anomalies in the meiotic germline process can lead to the production of abnormal spermatozoa [28]. Consequently, embryos with chromosomal abnormalities may develop. Studies show that men with sperm chromosomal abnormalities have a poorer prognosis for successful fertilization [29]. For instance, a study examining 131 ICSI cycles from couples with a history of RIF or multiple abortions confirmed that sperm aneuploidy significantly affects reproductive outcomes, particularly with respect to recurrent miscarriages and RIF [3,30].

## 5. Benefits of FISH Sperm Chromosomal Analysis

### 5.1. Benefits of Sperm Chromosomal Analysis for Genetic Counseling

Genetic counseling helps individuals understand and manage the psychological, medical, and familial impacts of hereditary predispositions to illness. A key component of genetic counseling involves compiling and analyzing a detailed three-generation family tree, which includes information on infertility, multiple miscarriages, stillbirths, consanguinity, intellectual disabilities, genetic disorders, birth abnormalities, and ethnic background. This comprehensive assessment is crucial for diagnosing underlying psychological issues and providing psychosocial support to men experiencing infertility. The timing of infertility disclosure is a critical aspect of the genetic counseling process [31].

Male infertility can result from genetic issues such as Y-chromosome microdeletion or balanced translocation. Klinefelter syndrome, characterized by the presence of an extra X chromosome (47,XXY), can lead to gonadal failure, impacting spermatogenesis and testosterone production [32]. Despite these challenges, successful sperm recovery from some 47,XXY males has been reported. When using intra-cytoplasmic sperm injection (ICSI), some studies suggest that the risk of aneuploidy in children may be higher than in naturally conceived children. Genetic counseling should address options and provide information on preimplantation and prenatal diagnosis [33].

Congenital bilateral absence or dysfunction of the vas deferens occurs in 1–2% of infertile males due to mutations in the cystic fibrosis transmembrane conductance regulator (CFTR) gene [34]. Genetic testing can identify heterozygous or homozygous pathogenic variants in the CFTR gene, which increases the risk of having children with severe forms of cystic fibrosis (CF). It is essential to offer CF carrier testing to partners to inform them of their risks as a couple. If a partner tests negative for CF, the risk remains low, but if they are a carrier, there is a potential risk of having a child with severe CF [35]. Genetic counseling should cover recurrence risk, clinical manifestations, available options, and patient resources. Men who sought genetic counseling for congenital bilateral absence of the vas deferens (CBAVD) and CF testing were generally more concerned about their reproductive potential than about health implications [31].

### 5.2. Preimplantation and Prenatal Genetic Diagnosis

Preimplantation genetic testing (PGT) offers an option for preventing the birth of affected offspring. The process involves an initial IVF/ICSI procedure, followed by a biopsy of embryonic cells, and then PGT to selectively transfer an unaffected embryo [36]. Increased frequencies of aneuploidy have been observed in embryos created through IVF/ICSI, which are then assessed using PGT. For patients with sperm abnormalities below 65%, IVF offers a reasonable chance of conception. A FISH study has indicated predictive value in FISH analysis [3], although other studies have produced contradictory results.

PGT plays a crucial role in identifying and eliminating chromosomally abnormal embryos, thereby facilitating viable pregnancies. However, the presence of chromosomal mosaicism in embryos raises concerns about the safety and accuracy of the testing. It is important to discuss the possibility of diagnostic inaccuracies and the options for prenatal diagnosis, such as chorionic villus sampling (CVS) and amniocentesis, during genetic counseling [37].

### 5.3. Male Infertility Linked to Chromosomal Abnormalities

The spermatozoon karyotype can be directly analyzed using the human sperm-hamster method, though obtaining metaphase chromosomes is challenging. Fluorescence in situ hybridization (FISH), developed in the 1990s, offers advantages such as interphase detection, increased statistical power, high sensitivity, and specificity for assessing sperm chromosome number anomalies. FISH enables precise identification of chromosomal numbers [9].

Currently, the extent of aneuploidy across each of the 23 chromosomes in sperm cells from normozoospermic males is not well understood. Most studies focus on a limited number of chromosomes, particularly chromosomes 13, 18, 21, and sex chromosomes [38]. Due to space constraints within the sperm and limitations related to the fluorescent molecules and the cost of probes, FISH cannot detect all chromosomes in a single sperm nucleus. Human sperm exhibit aneuploidy rates ranging from 0.03% to 0.47% for each of the 18 chromosomes analyzed [39]. This indicates that sperm aneuploidy is relatively uncommon and requires extensive analysis to detect, which poses challenges for researchers. Nonetheless, additional chromosomal analysis can provide insights into the overall occurrence of anomalies. Research often focuses on specific chromosomes in cases of trisomy to evaluate embryo viability [40].

Common chromosomal number aberrations in normozoospermic males include disomy, nullisomy, and diploidy. Disomy, especially involving chromosomes 13, 18, 21, and XY, is the most extensively studied anomaly due to its higher occurrence in live births [41]. Mean disomy frequencies from control donor data across 49 sperm FISH examinations are consistent with ongoing research. The mean disomy frequencies for the remaining 19 chromosomes ranged from 0.08% to 0.32% [42]. Reports of nullisomy in the normal population show a mean frequency of 0.51% for sex chromosomes and chromosomes 13, 18, and 21. The incidence of nullisomy for chromosome 21 aligns with current findings, while chromosome 13 shows a slightly higher rate [13,39]. For chromosomes 1, 2, 4, 6, 7, 9, 11, 15, 17, and 19, mean nullisomy frequencies are roughly two to three times higher, while frequencies for chromosomes 8, 10, and 12 are much lower. Disomy is found to be twice as common as the average incidence per chromosome [42]. According to Bell et al., chromosomal losses are more frequent than gains, with a ratio of 2.4:1 [43].

The frequencies of disomy and nullisomy per chromosome were used to calculate the overall frequency of aneuploidy for each chromosome [43]. Mean frequencies for chromosomes 4, 6, 7, 8, 9, 10, 11, 12, 13, 17, 18, 21, and XY were similar to those reported by M.G. Pang and Tang et al. in previous studies [44,45]. However, the frequency observed for chromosome 18 in the current investigation was notably lower. By analyzing chromosomes from all donors, the research team determined the frequencies of disomy, nullisomy, and overall aneuploidy for each donor. Neusser et al. also observed disomy frequencies for 23 chromosomes in three normal controls [46].

Calculating total aneuploidy involves summing the frequencies of disomy and nullisomy. However, if the frequencies vary significantly, this method may not be accurate. Templado and colleagues reported a total disomy rate of 2.26% for the normal population, which is comparable to the recent finding of 2.69% for 18 chromosomes [47]. Earlier studies estimated a total disomy rate ranging from 5.38% to 4.5% by summing frequencies. A fourth study reported 84%, nearly double the current estimate, by combining both disomy and nullisomy frequencies. Summing both frequencies is generally preferred due to variations in perinucleation rates between chromosomes and types of abnormalities. This study is the first to provide a mean frequency of total numerical aberration per donor for 23 chromosomes, at 11.63%. Further research is needed to validate these findings and understand the range of variation [39].

Chromosome abnormalities are more prevalent in infertile males with low semen parameters [48]. Increased aneuploidy rates are also observed in males from couples with repeated miscarriages and in fathers of patients with Turner syndrome or Down syndrome [49,50]. Screening high-risk populations can benefit from clinical diagnosis and counseling. Identifying individuals with a higher likelihood of producing abnormal spermatozoa is known as aneuploidy screening. The 95% confidence interval shows how much an individual’s rates might differ from those of a normal fertile population. García-Mengual et al. reported upper boundaries for disomy and nullisomy frequencies, with higher nullisomy boundaries observed for chromosomes 16 and 22 compared to this study. Due to varying settings, FISH results may differ between studies. Reliable screening of high-risk groups requires comparing individual rates to upper boundaries reported in relevant research [12].

Approximately 6% of males of reproductive age are affected by male factor infertility. FISH is useful for assessing aneuploidy and is recommended for males with repeated miscarriages. It also supports genetic and reproductive counseling for affected couples. With its growing clinical application, FISH may advance preimplantation genetic screening, diagnosis, and treatment [10].

Male infertility, affecting 12% of those of reproductive age, can often be addressed with assisted reproductive technologies like IVF. Severe cases may require ICSI. DNA damage or extrinsic factors can lead to errors in stem cell division, contributing to male factor infertility [51]. Although the long-term effects of ICSI are still largely unknown, it can increase the incidence of aneuploidies. A significant drawback of ICSI is the method of sperm selection [52]. Sperm used in FISH studies cannot later be used for adoptive cell transfer (ART). Sperm selection limitations, as shown by FISH procedures, highlight that couples with normal semen characteristics but frequent miscarriages or unsuccessful IVF treatments might not fully consider sperm aneuploidies. FISH cytogenetic analysis can aid in identifying the causes of infertility [10].

## 6. Novel Methodology

Despite significant advances in clinical genetics, up to 80% of males with infertility still struggle to receive a definitive diagnosis. This highlights the need for continued research. The complexity of spermatogenesis and the diverse phenotypes of infertile men make it challenging to identify and treat multiple genetic targets effectively. However, recent advancements in methods and technologies offer hope for improving both the diagnosis and treatment of infertility [53].

### 6.1. Next-Generation Sequencing and Genome Microarrays

Advancements in genetic testing have led to the development of new population-based experimental techniques, enhancing our understanding of the genetic causes of diseases [54]. The successful sequencing of the human genome in the early 2000s has made extensive databases of single nucleotide polymorphisms (SNPs) available, facilitating large-scale genome-wide association studies (GWAS) aimed at identifying genetic differences between populations [55]. Large-scale genome-wide association studies (GWAS), which are observational studies aimed at identifying genetic differences between populations, have been carried out using these databases over the last 15 years. These data are helpful to identify candidate genes for more research. The two main categories of genomic characterization techniques are NGS and microarray methods [56,57].

Genomic microarrays analyze complementary DNA strand binding patterns from cells of interest against probes on the array, enabling the simultaneous and accurate genotyping of thousands to millions of genomic regions. Commercially available and bespoke assays have identified genes linked to specific infertility etiologies. For example, a 250K SNP array revealed that mutations in the DPY19L2 gene were associated with globozoospermia, leading to further investigation into the role of this protein in sperm [58]. Additionally, copy number variants (CNVs), or DNA segments with different numbers of repeats among individuals, are studied using fluorescently labeled DNA in comparative genomic hybridization (CGH). A microarray is used to test the hybridization of DNA samples from a case and control using labels that differ in color. It is feasible to ascertain the relative amount of complementary DNA between the two participants by observing the colors of each probe in the array [59]. Aside from identifying previously unknown etiologies, CGH-based CNV helped diagnose established ones. A unique CGH microarray was created by Yuen et al. in 2014, and while it cost almost twice as much as PCR, it could successfully identify YCMD at greater resolutions [60].

Next-generation sequencing (NGS) has revolutionized genome sequencing by dramatically reducing costs and increasing efficiency. The initial NGS platforms introduced in the mid-2000s achieved a 50,000-fold reduction in cost, with subsequent improvements further enhancing efficiency [61]. NGS is now employed in three key applications: whole exome sequencing (WES), targeted sequencing (TS), and whole genome sequencing (WGS). Targeted sequencing utilizes disease-specific gene arrays to simultaneously sequence relevant genes, which is particularly valuable in the context of infertility as it can screen for single-gene mutations, microdeletions, and chromosomal abnormalities.

Despite the significant clinical promise of microarrays and NGS in identifying potential causal genetic variations, their clinical application has been limited [62] (Table 1). Challenges include small sample sizes, unclear clinical significance of identified genes, and inconsistent validation study outcomes. A comprehensive review by Oud et al. found moderate evidence for only 92 genes associated with male infertility out of 521 gene-disease associations assessed [63]. This underscores the need for further confirmatory research. Advances in genomics and bioinformatics will facilitate more effective analysis of future genetic studies.

### 6.2. Epigenetics for Sperm Analysis

Genetic epigenesis involves changes in DNA that affect cell differentiation without altering the DNA sequence itself. These epigenetic modifications can influence sperm and gene expression in somatic cells. Reactive oxygen species and oxidative stress may alter the epigenetic landscape of sperm DNA, potentially impacting gene transcription during embryogenesis and contributing to miscarriage [64]. Despite this, using epigenetic modifications as markers for male factor infertility is challenging due to their often subtle biological effects [65].

Specific epigenetic changes, such as the hypomethylation of the H19 gene and the hypermethylation of the MEST and SRNPN genes, have been associated with sperm abnormalities. These changes may affect gene transcription patterns and DNA methylation, potentially influencing the success rates of intrauterine insemination (IUI) and in vitro fertilization (IVF) [66]. For example, in embryos undergoing preimplantation genetic screening, patients with a history of oligoasthenoteratozoospermia (OAT) have shown increased DNA methylation and altered gene expression. While genetic screening can identify male infertility following IVF, it remains unclear whether these epigenetic changes indicate a distinct underlying condition or are a direct cause of infertility and subfertility. Examining DNA methylation, which remains stable throughout spermatogenesis, can help in detecting male infertility [13].

Environmental pollutants, particularly those from plastics, can lead to heritable aberrant DNA methylation in sperm. Additionally, reduced sperm quality has been linked to conditions such as diabetes and obesity [67]. Currently, the only commercially available screening method for epigenetic modifications in sperm is Seed, although more companies are expected to develop similar tests. Epigenetic modifications occurring after transcription may contribute to male infertility. MicroRNAs and other short non-coding ribonucleic acids (sRNAs) play crucial roles in spermatogenesis. Changes in the sRNA profile of sperm due to environmental stressors may affect embryonic development and offspring phenotype. Understanding these modifications could help in avoiding negative outcomes associated with sperm used in assisted reproduction technologies [68].

### 6.3. Proteomics

Protein structure and function vary between different cells, and these variations are studied through proteomic analysis. In the context of male fertility, the protein composition of ejaculate, which originates from either the testis or the epididymis, is complex [69]. Mass spectrometry is used to identify proteins in various components of sperm, including the sperm membrane, different sperm sections, mitochondria, and seminal plasma. Changes in a single protein or its downstream effects can contribute to infertility phenotypes [70].

Proteomic patterns in semen have been linked to repeated IVF failures [71]. The complexity of analyzing semen proteome as a diagnostic for infertility stems from environmental influences and variability. For example, mice exposed to endocrine disruptors exhibit proteins associated with cell death, highlighting the impact of external factors on protein expression [72]. Despite this, identifying consistently altered proteins across different infertility phenotypes remains challenging.

Recent studies have identified distinct proteome fingerprints in men with normal semen characteristics using advanced quantitative proteomic techniques. Nevertheless, the current categorization methods may not fully account for the considerable heterogeneity observed among infertile males with aberrant semen characteristics [73].

Proteomics is increasingly important in male infertility research. For instance, specific proteins in fertile males with high reactive oxygen species (ROS) levels may be elevated to counteract ROS damage and maintain fertility [74]. In patients with asthenozoospermia, heat shock protein A4L is downregulated, leading to reduced sperm motility and impaired sperm-oocyte penetration. Future proteomic research may lead to the development of biomarkers for male infertility by targeting specific proteins in seminal plasma and sperm [75].

### 6.4. Metabolomics

Metabolomics is the study of metabolic byproducts in cells resulting from gene expression [76]. ROS production can result from epigenetic modifications brought on by oxidative stress in sperm. Infertile men who have elevated ROS levels also have poor sperm motility, concentration, and shape [77]. ROS production can stem from epigenetic modifications caused by oxidative stress in sperm.

One prominent application of metabolomics in this field is the proteomic analysis of ROS in semen, which has been explored as a potential indicator of male infertility. For instance, Raman spectroscopy has been used to study spermatogenesis in patients with non-obstructive azoospermia (NOA). Additionally, a different study analyzed seminal plasma from NOA patients undergoing testicular sperm extraction (TESE) using untargeted metabolomics profiling with gas chromatography-mass spectrometry (GC-MS). These findings could influence pre-operative screening and counseling for NOA patients [78].

Researchers are investigating the metabolic byproducts of spermatogenesis as potential biomarkers of male infertility [79]. Metabolite profiling has revealed significant changes in the metabolism of amino acids, energy, and nucleosides in infertile men, suggesting its potential for diagnostic purposes [80]. Accurate markers for male infertility may emerge from ejaculate metabolic profiling, offering minimally invasive diagnostic options. However, further research is needed to explore the therapeutic applications of semen sample [81].

## 7. Conclusions and Future Directions

Genetic analysis has changed dramatically over the years, providing a range of advanced methods that go beyond and supplement conventional Fluorescence In Situ Hybridization (FISH). Each technique has unique strengths, contributing to a deeper understanding of genetic and epigenetic factors in sperm analysis. Integrating these advanced methods allows for more accurate diagnoses and personalized treatment plans in assisted reproductive technologies. The advancements in reproductive genomics provide renewed hope for individuals facing infertility by enhancing diagnostic precision and tailoring interventions more effectively.

## Figures and Tables

**Table 1:** An overview of Fluorescence In Situ Hybridization (FISH) and alternative genetic analysis techniques, highlighting various methodological approaches and their applications in identifying genetic abnormalities.

Alternative Methods for FISH	Methodology
**Polymerase Chain Reaction** **(PCR)** ^1^ Quantitative PCR (qPCR) ^2^ Multiplex PCR ^3^ Digital PCR (dPCR)	^1^ Enables the identification of gene mutations, deletions, or amplifications by quantifying particular DNA sequences. ^2^ Allows numerous targets to be amplified simultaneously in a single reaction, which is helpful for detecting various genetic abnormalities. ^3^ Provides target DNA molecule absolute quantitation without requiring reference standards.
**Next-Generation Sequencing** **(NGS)** ^1^ Whole Genome Sequencing (WGS) ^2^ Whole Exome Sequencing (WES) ^3^ Targeted Gene Panels	^1^ Allows the diagnosis of a broad variety of genetic abnormalities by offering a thorough examination of the whole genome. ^2^ Emphasizes the genome’s protein-coding regions, which are frequently the most important in terms of function. ^3^ Sequencing of a particular gene set known to be related to a given disease or condition.
**Comperative** **Genomic Hybridization** **(CGH)** Array CGH	Provides details on the deletions and duplications of significant DNA sequences by identifying and measuring copy number variations (CNVs) throughout the genome.
**Single Nucleotide Polymorphism (SNP) arrays**	Linkage analysis and the identification of genetic variations can both benefit from these arrays’ ability to detect differences at single nucleotide sites throughout the genome.
**Southern Blotting**	An approach for identifying particular DNA sequences in a sample of DNA. Finding significant insertions, deletions, and other structural changes is helpful.
**Karyotyping**	Specializing in significant chromosomal abnormalities like translocations, inversions, deletions, and duplications, traditional karyotyping offers a visual assessment of chromosomes.
**Microfluids and Lab-on-a-Chip Technologies**	Emerging technologies that, frequently with faster throughput and reduced sample consumption, enable the microscale study of genetic material.
**Single Cell Genomics**	Methods such as single-cell DNA sequencing and single-cell RNA sequencing (scRNA-seq) can yield comprehensive data at the level of individual sperm cells.

## Data Availability

Data is unavailable due to privacy or ethical restrictions.
